# Robotic instrumentation: Evolution and microsurgical applications

**DOI:** 10.4103/0970-1591.70580

**Published:** 2010

**Authors:** Sijo J. Parekattil, Michael E. Moran

**Affiliations:** University of Florida, Gainesville, FL

**Keywords:** Evolution, history, instrumentation, microsurgery robotic

## Abstract

This article presents a review of the history and evolution of robotic instrumentation and its applications in urology. A timeline for the evolution of robotic instrumentation is presented to better facilitate an understanding of our current-day applications. Some new directions including robotic microsurgical applications (robotic assisted denervation of the spermatic cord for chronic orchialgia and robotic assisted vasectomy reversal) are presented. There is a paucity of prospective comparative effectiveness studies for a number of robotic applications. However, right or wrong, human nature has always led to our infatuation with the concept of using tools to meet our needs. This chapter is a brief tribute to where we have come from and where we may be potentially heading in the field of robotic assisted urologic surgery.

## INTRODUCTION

The history of robotics in urology is rich and diverse. From the earliest beginnings of urology in the time of Hippocrates[1](aside the references to urine diagnosis contained in Babylonian, Egyptian, and Indian medicine), there has been continued development of instrumentation to assist the urologist in the diagnosis and treatment of urologic disorders. This chapter will focus on the evolution and development of robotic assisted instrumentation in urology. A detailed timeline of the development of key robotic instrumentation advances is presented [Table 1]. A review of some novel robotic assisted microsurgical applications in urology is briefly covered as well. An understanding of the evolution of our instrumentation may assist us in the further development of robotic tools for the future.

**Table 1:** Historical timeline of the evolution and development of robotic instrumentation in urology

Year	Device / Instrument	Group	Application
1961	UNIMATE	Unimation, Inc., USA	First industrial robot in USA
1967	Versatron	American Machine and Foundary	First industrial robot in Japan
1978	Unimate PUMA	Unimation, Inc., USA	Electric motor based programmable miniaturized version of unimate
1979	Definition of “Robot” established	Robot Institute of America	Reprogrammable, multifunctional manipulator designed to move materials, parts, tools, or specialized devices through various programmed motions for the performance of a variety of tasks
1980s	Telepresence surgical system	Stanford Research Institute (SRI) & National Aeronautics and Space Administration (NASA) Ames Research Center	Anastomosis of femoral arteries
1980s	SRI Green Telepresence Surgical System	SRI & US Department of Defense	Open surgery - military applications
1980s	SRI Green Telepresence Surgical System	Bowersox and Cornum	Nephrectomy, cystotomy closure, ureteral anastomosis - porcine model
1988	PUMA	Imperial College, London, UK	Transurethral prostate resection
Late 1980s	SARP (Surgeon Assistant Robot for Prostatectomy)	Imperial College, London, UK	Transurethral prostate resection
1991-1997	PROBOT (Robot for prostatectomies)	Harris *et al*.	Transurethral prostate resection (TURP)
1990s	ARTEMIS system	Schurr *et al*. (Germany)	Procedures in animal models
1993	HERMES	Computer Motion, Goieta, CA	Voice controlled integration of operative room components
1993	AESOP (Automated Endoscopic System for Optimal Positioning)	Computer Motion, Goieta, CA	Laparoscopic abdominal surgery
1997	Prototype of da Vinci surgical system	Intuitive Surgical, Sunnyvale, CA (founded 1995)	Laparoscopic surgery
Late 1990s-2001	URobot	Nanyang Technological University (NTU - Singapore)	TURP, prostate biopsy, brachytherapy seed placement
1997-2002	PAKY (Percutaneous access to the kidney)	Johns Hopkins University & Medical Center	Percutaneous access to kidney
1998	Surgeon programmable urological device (SPUD)	NTU & Dornier Asia medical	TURP, prostate biopsy, brachytherapy seed placement
2000	da Vinci Surgical System	Intuitive Surgical, Sunnyvale, CA	Laparoscopic surgery
2001	Zeus Surgical System	Computer Motion, Goleta, CA	Laparoscopic surgery
2003			Merger of Intuitive Surgical & Computer Motion
2004	Robotic system forTRUS prostate	Johns Hopkins University	Trans-rectal ultrasound guided biopsy of prostate
2006	Robotic system for TRUS guided brachy therapy	Johns Hopkins University	Trans-rectal ultrasound guided brachytherapy
2006	Type S da Vinci robotic system	Intuitive Surgical, Sunnyvale, CA	Laparoscopic surgery
2009	Type Si da Vinci robotic system	Intuitive Surgical, Sunnyvale, CA	Laparoscopic surgery
2009	Robotic Doppler Micro Probe	Vascular Technology, Nashua, NH	Doppler for vascular identification during robotic microsurgical procedures

## METHODS

A review of published literature, commercial patents and expert opinions were garnered to prepare a historical timeline for the evolution of robotic instrumentation in the field of urology [Table 1]. A brief presentation of new robotic microsurgical instrumentation and new applications was then prepared.

## HISTORICAL TIMELINE

Table 1 presents a historical timeline of the development of robotic instrumentation in urology.

### 

#### 1961 - Unimate

Isaac Asimov first used the word ‘robotics’ in a short story, “Runaround” published in 1942. One of the first robots Asimov wrote about was a robotherapist - a communication liaison device between man and machine. In 1956, a historic meeting occurred between George C. Devol, a successful inventor and entrepreneur, and engineer Joseph F. Engelberger. Over cocktails, the two discussed the writings of Isaac Asimov. They formulated the beginnings of the first commercially viable robot. They persuaded Norman Schafler of Condec Corporation in Danbury to invest in their venture. Engelberger started the first commercial company to make robots called ‘Unimation’ (universal automation). Devol wrote the necessary patents. Their first robot was called the ‘Unimate’. This resulted in Engelberger being called the ‘father of robotics.’ The first Unimate was installed at a General Motors plant to work with heated die-casting machines. The goal of Unimate was to complete tasks that would be considered otherwise unsafe and difficult for humans to perform. Most Unimates were used for die castings extractions. They were also utilized for spot welding on auto bodies. Such tasks were considered particularly hateful jobs for humans. The use of the Unimate for these applications was a huge commercial success. The robots worked reliably and saved money by replacing manual labor.

This was the beginning of a very productive robotic industrial revolution that spawned the modern usage of robots in many of our factories. The Unimate was applied to a variety of other tasks, such as loading and unloading machine tools. Ultimately Westinghouse acquired Unimation and the entrepreneurs’ dream of wealth was achieved. Unimation is still in production today, with robots for sale. The robot idea was hyped to the skies and became high fashion among senior management. Chief executive officers of large corporations bought them, for about $100,000 each, just to put into laboratories to “see what they could do”.[2] In fact, these sales constituted a large part of the robot market. Some companies even reduced their return on investment criteria for investment for robots to encourage their use.

#### 1967 - Versatron

The first industrial robot was introduced in Japan in 1967. It was a Versatron robot from American Machine and Foundary (AMF). The following year, Kawasaki licensed the hydraulic robot designs from Unimation and started production in Japan. From that time onwards, Japan has rapidly become the global leader in the design, development, and distribution of robots of all types (particularly industrial). The whole of the now European Union did bypass Japan in the number of industrial robotic installations in 2001. But, no single country even comes close to Japan in the number of robotic applications. The International Federation of Robotics estimates that Japan (approximately the size of California) installed three times the number of industrial robots than did the U.S. in 2001 (28,369 vs. 10,824). Germany, which is also a very industrialized country, installed 12,524 robots in the same year. According to the World Fact Book 2002, Japan possesses 410,000 of the world’s 720,000 “working robots.”

#### 1978 – Puma

The Puma (Programmable Universal Machine for Assembly) robot is developed by Unimation. This device utilizes electric motors and is a much smaller and more versatile version of the unimate robot.

#### 1979 - What is a robot?

The Robot Institute of America sets a definition of what a robot is: “A reprogrammable, multifunctional manipulator designed to move materials, parts, tools, or specialized devices through various programmed motions for the performance of a variety of tasks”.

#### 1980s - Green SRI telepresence surgical system

The United States Department of Defense had long been interested in the development of front-line methods of improving care to injured soldiers. Life-threatening injuries occurring immediately during battle might be salvageable if surgical care could be instantly instituted. In addition, after George Bush’s announcement of the United States’ intention of getting a man on Mars, the National Aeronautics and Space Administration (NASA) Ames Research Center began to fund proposals for the eventual need for possible surgical intervention on astronauts remote from a hospital.[3] A team of investigators led by Michael McGreevey and Stephen Ellis began to investigate 19,861 computer-generated scenarios that could be perceived on head-mounted displays (HMDs).[4] To this team, eventually came Scott Fisher who added 3D audio and came up with the concept of “telepresence.” This was the notion that one person could be projected with the immersive experience of another (real or imaginary). Joseph Rosen, a plastic surgeon at Stanford University began to experiment with Philip Green from Stanford Research Institute (SRI) to develop dexterity-enhancing robots for telemanipulation.[5] These two teams would eventually collaborate, and together Joe Rosen and Scott Fisher produced the fundamentals of telepresence surgery. This combined the dexterity-enhancing robotics of Green and the “virtual reality” systems of NASA for an immersive surgical experience. The initial systems conceived that the surgeon would be in a helmeted immersive sight/sound environment wired electronically to “data gloves” that would digitally tract the surgeon’s motions and reproduce them at remote robotic instruments. The notion of the data glove came from Jaron Lanier, a computer scientist interested in Virtual Reality. The initial targeted surgery was on the hand and vascular anastomosis.

Many of the initially designed features of Green’s Telepresence System were at the time unworkable from an engineering standpoint. The HMD was subsequently replaced with monitors, and the data gloves were replaced with handles for controllers at the surgeon’s console. Since the imperative at this time was for space and/or military application for acute surgical care, the end-effectors were substantially similar to open surgical instruments. This was all occurring in the late 1980s. By 1989, then Colonel Richard Satava stationed at Silas B. Hayes Army Hospital in Monterey became involved in this project and more Federal aid became available. Serendipitously, that same year found Jacques Perrisat of Bordeaux presenting on the technique of laparoscopic cholecystectomy at the Society of American Gastrointestinal Endoscopic Surgeons (SAGES) in Atlanta. Upon returning from this meeting, the team of investigators began to consider developing a system that could be applied to minimally invasive laparoscopic surgery. Satava presented a videotape of a bowel anastomosis using the telepresence surgery system to the Association of Military Surgeons of the United States The results of this single demonstration of this technology resulted in a July 1992 Defense Advanced Research Projects Agency (DARPA) grant for further investigation and development. In addition, Satava became the program manager for Advance Biomedical Technologies to aid funding of technologically advanced projects. With the funding now possible, by 1995, the robotic system was in prototype mounted into an armored vehicle (the Bradley 557A) that could “virtually” take the surgeon to the front lines and immediately render surgical care to the wounded, called MEDFAST (Medical Forward Area Surgical Team).[6] The technology caught the attention of Alan Alda (aka Hawkey Pierce from the T.V. drama M.A.S.H.) now the voice of Discovery Channel who filmed a piece on this technology.

#### 1988 – PUMA for trans-urethral prostatectomy

**Figure 1:**
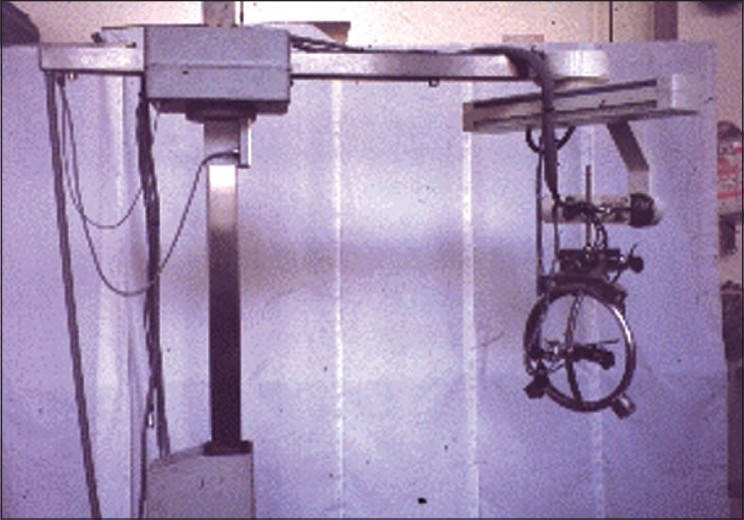
The Probot robotic device for trans-urethral resection of the prostate

Initial experiments with a six-axis unimate PUMA to perform trans urethral resection of the prostate are performed successfully at Imperial College, London.[7] Further developments in this technology lead to the development of the SARP (Surgeon Assistant for Prostatectomy) in 1990. This utilized a motorized version of the manual frame used in the previous robot. It was used on a patient successfully on 25 March 1991 in Shaftesbury Hospital, Institute of Urology, London, UK. This was perhaps the world’s first robotic surgery on the prostate. Further development on SARP, led to the creation of PROBOT (robot for prostatectomies – Figure 1).[8]

#### 1990s – ARTEMIS

**Figure 2:**
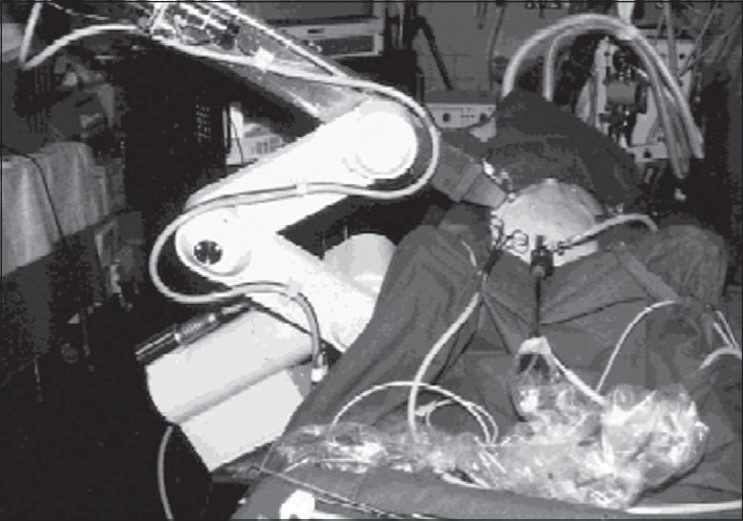
The ARTEMIS robotic system for minimally invasive surgery. (With permission from Schurr *et al*., Robotics and telemanipulation technologies for endoscopic surgery, Surg Endosc, 2000;14:375– 81)

In Germany, Schurr *et al*.,[9] developed the ARTEMIS system (Advanced Robotic Telemanipulator for Minimally Invasive Surgery). This was probably the first robotic system that delivered six degrees of freedom for surgery [Figure 2]. However, the project failed to garner continued funding and was eventually terminated.

#### 1993 – HERMES and AESOP

**Figure 3:**
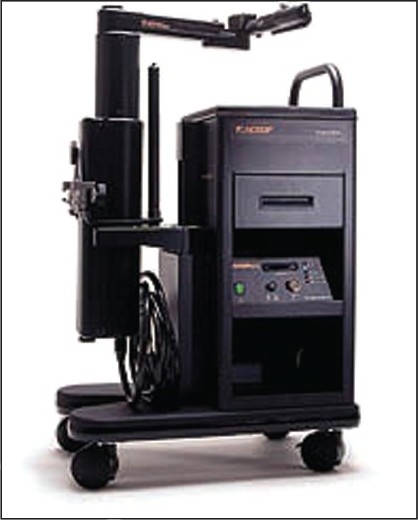
The AESOP system, Automated Endoscopic System for Optimal Positioning (Computer Motion, Goleta, CA)

**Figure 4:**
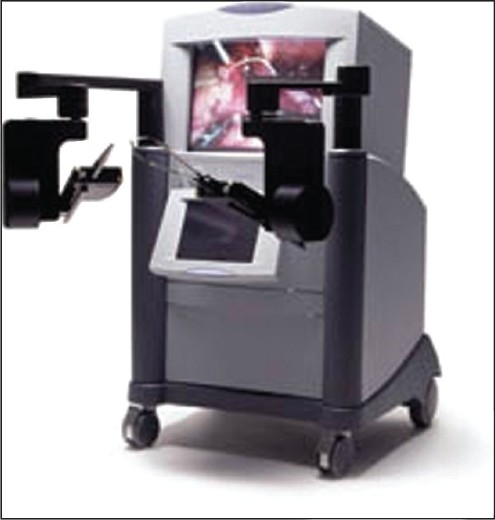
The ZEUS robotic system, Master-slave device (Computer Motion, Goleta, CA)

In 1993, Yulyn Wang, Ph.D. from the University of California, Santa Barbara developed software for control motion of robotic systems and founded a company called Computer Motion. Wang succeeded in developing a robotic camera holder called Automated Endoscopic System for Optimal Positioning (AESOP). He became interested in complete robotic surgery and obtained DARPA (Defense Advanced Research Projects Agency) funding and money from entrepreneurs to develop ZEUS, a modular robotic system to be integrated with AESOP [Figure 3]. HERMES was the integrated operating room control system that allowed the complete integration of Computer Motion’s robotic system.[10] In 2001, a device combining both the AESOP and HERMES was developed by Computer Motion, the ZEUS robotic system [Figure 4]. This was a master-slave device that allowed the surgeon to be positioned at a console and control a separate robotic slave device.

#### 1990-2001 – URobot

The SARP and PROBOT devices (from Imperial College, London) were further developed at the Nanyang Technological University in Singapore to develop the URobot in 1991. This device was utilized for trans-urethral HIFU (High Intesity Foucsed Ultrasound), brachytherapy, needle prostate biopsy and laser resection of the prostate.[11,12] A collaboration with Dornier Asia Medical Systems led to the creation of the SPUD device (Surgeon Programmable Urological Device). However, no further publications have been presented yet on this device.

#### 1997-2002 – PAKY

**Figure 5:**
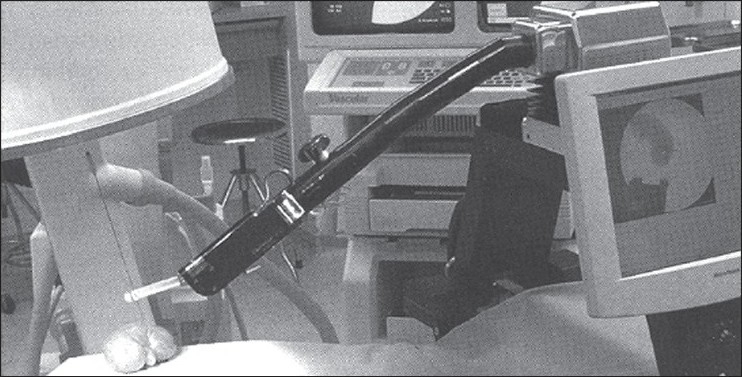
The PAKY robotic system (With permission from Kim *et al*., The PAKY, HERMES, AESOP, ZEUS, and da Vinci robotic systems, Urol Clin N Am 31, 2004, 659-669)

The Johns Hopkins Medical Center has been involved in the development of a robotic system to perform Percutaneous Access to the Kidney (PAKY – Figure 5).[13–15] In early porcine models, the device achieved an accuracy of 83% in accessing the desired calyx. In live animal trials the accuracy dropped to 50% due to real-time changes in tissue and needle deflection in this scenario. Continued development of this system is ongoing.

#### 1995-2000 - da Vinci surgical system

In 1995, Frederic H. Moll, M.D. (a successful entrepreneur), Rob Younge (an engineer) and John Freund (an MBA from Harvard) became interested in the potential of the “telepresence” work from SRI. They created a start-up company called Intuitive Surgical.[16] The device comprised three main components: 1) a master-slave software-driven system that provided control of seven-degree-of-freedom robotic instruments, 2) a three-dimensional immersive vision system, and 3) a sensor-based safety monitoring system to continuously reassess the device’s performance to maximize patient safety.

**Figure 6:**
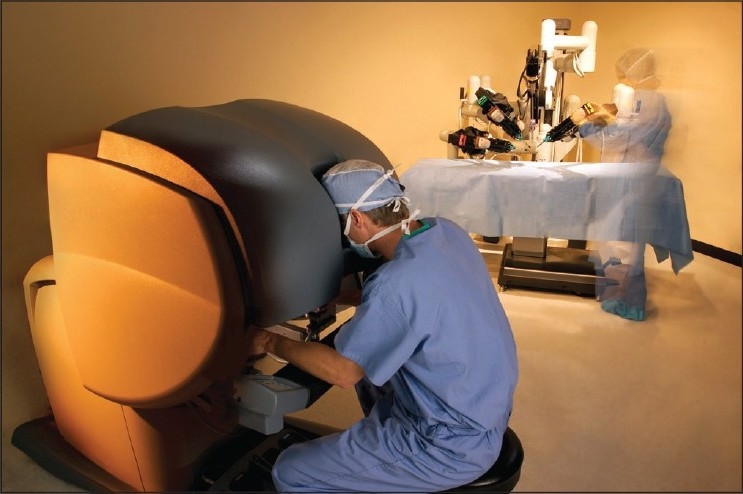
The original da Vinci robotic system (With permission from Intuitive Surgical Inc., Sunnyvale, CA)

The first prototype was tested in March 1997. By April 15, 1997 the first robotic surgery was performed by Jacques Himpens and Guy Cardiere of Brussels, Belgium: a robotic cholecystectomy.[17] The first 200-patient trial was completed on cholecystectomy and Nissen fundoplications leading to Food and Drug Administration (FDA) approval of this robotic system in July 2000 [Figure 6]. In December 2002, the FDA also approved the use of the next generation da Vinci System with the addition of a fourth robotic arm to the tower. The use of the robot in urology rapidly advanced with applications in prostatectomy, nephrectomy, partial nephrectomy and cystectomy.

#### 2003 – Merger of Computer Motion and Intuitive Surgical

In 2003, Computer Motion and Intuitive Surgical merged to form Intuitive Surgical, Inc. The da Vinci platform development continued, however, the ZEUS line of products was ended.

#### 2004 – John Hopkins Robotic TRUS prostate device

**Figure 7:**
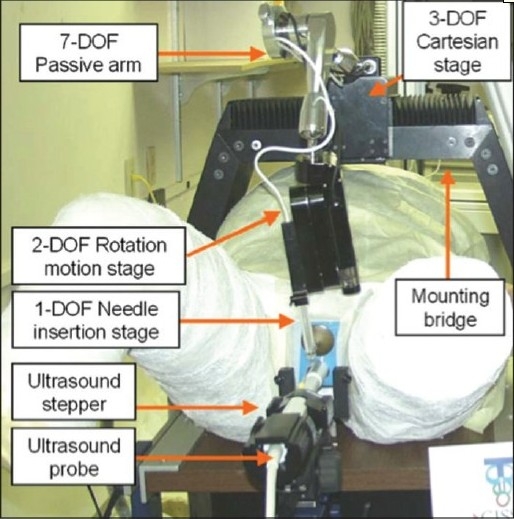
The John Hopkins Robotic TRUS brachytherapy system (With permission from Fichtinger *et al*., Robotically assisted prostate brachytherapy with transrectal ultrasound guidanced Phantom experiments, Brachytherapy 2006;5:14– 26

Schneider, Okamura and Fichtinger initially develop a robotic system to perform transrectal ultrasound (TRUS) image-guided biopsies of the prostate in 2004.[18] Continued development on this device with Dr. Dan Stoianovici lead to the use of this device for brachytherapy [Figure 7].[19] Preliminary experimental results for this device showed that the average placement error for needles in the prostate was only 2 mm (transverse error) and 2.5 mm (sagittal error). Continued development of this platform is ongoing and its use to provide real-time intra-operative mapping of the neurovascular bundles during robotic assisted radical prostatectomy is being investigated.

#### Current robotic surgical platform and applications

**Figure 8:**
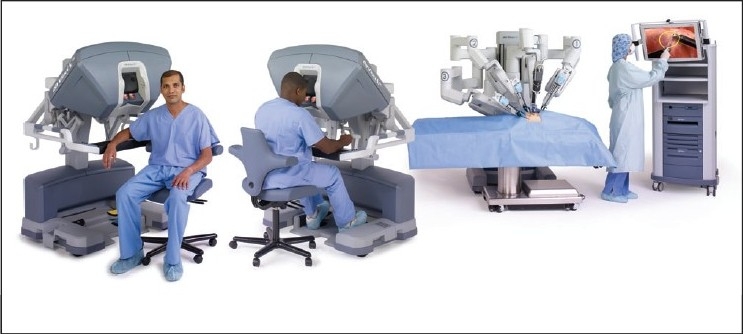
The Si da Vinci robotic system (With permission from Intuitive Surgical Inc., Sunnyvale, CA)

Intuitive Surgical (Sunnyvale, CA) now offers an enhanced four-arm DaVinci type S or Si robotic system [Figure 8] with High-Definition digital visual magnification that allows for greater magnification than the standard robotic system (up to 10-15x). The enhanced magnification capability allows the surgeon to position the camera 6-7 cm away from the operative field to avoid any local tissue effects from the heat emitted from the camera lighting (this was a problem with the older system, where the camera had to be placed within 2-3 cm of the operative field for microsurgery). This new system allows greater range of motion and better microsurgical instrument handling. The additional fourth arm has improved range of motion and positioning capabilities to provide the microsurgeon with one additional tool during procedures. The new Si system also allows for dual surgeons to operate via dual consoles that may control one robotic system. This allows a primary surgeon to guide a trainee surgeon through robotic procedures.

#### New robotic microsurgical advances in urology

**Figure 9:**
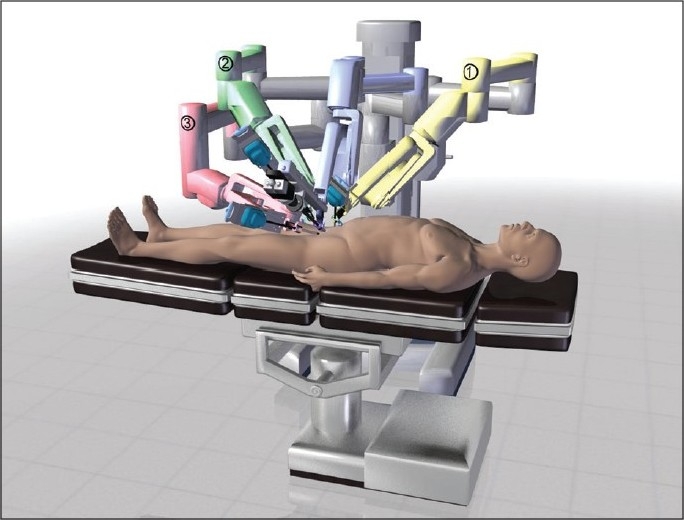
The da Vinci robotic system positioning for microsurgical cases

The da Vinci platform is now being utilized for robotic assisted microsurgical procedures in urology such as: vasectomy reversal, sub-inguinal varicocelectomy and denervation of the spermatic cord. The robot is positioned from the right side of the patient for microsurgical cases as illustrated in Figure 9. The platform is used to complete the microsurgical components of the surgery in lieu of an operating microscope. There are a few enhancements and devices that have been developed that may take robotic microsurgical applications further. These are presented below with a brief introduction of some new applications in urologic microsurgery.

#### Enhanced 100× digital visual magnification

The miniaturization and development of advanced digital microscopic cameras (100×-250×) allows even greater magnification than the standard robotic (10-15×) and microscopic (10-20×) magnification in use at this time. Our group is currently involved in clinical trials of a 100× digital camera (Digital Inc, China) that can be utilized via the TilePro™ DaVinci S or Si robotic system (Intuitive Surgical, Sunnyvale, CA) to allow the surgeon to toggle or use simultaneous 100× and 10-15× visualization. This provides the surgeon with unparalleled visual acuity for complex microsurgical procedures.

#### Robotic doppler flow probe

**Figure 10:**
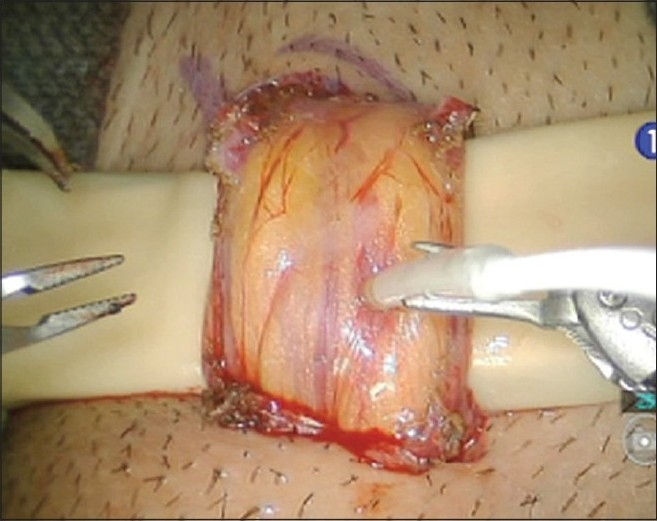
The new micro Doppler probe for robotic microsurgery (used here to identify testicular artery in the spermatic cord during varicocelectomy)

A recent study by Cocuzza *et al*.,[20] has shown that the systematic use of intra-operative vascular Doppler ultrasound during microsurgical subinguinal varicocelectomy improves precise identification and preservation of testicular blood supply. During robotic microsurgical cases, the standard Doppler probe has to be held by a surgical assistant and cannot be manipulated readily with the robotic graspers. Vascular Technology Inc (Nashua, NH) has developed a new Micro-Doppler flow probe (MDP) designed specifically for use with the robotic platform [Figure 10]. This new probe allows for easy manipulation of the probe with the fourth arm and allows the surgeon to perform real-time Doppler monitoring of the testicular artery during cases such as robotic assisted microscopic varicocelectomy (RAVx) and robotic assisted microscopic denervation of the spermatic cord (RMDSC). This allows the surgeon to hear the testicular artery flow while dissecting out the veins and nerves with the other two robotic arms.

The MDP was evaluated in eight robotic microsurgical cases (three subinguinal varicocelectomies and five spermatic cord denervation procedures) for efficacy in testicular artery localization and ease of robotic grasper maneuverability. It was effective in identifying all testicular arteries within the spermatic cord in all cases. Due to the compact size of the MDP, maneuverability using the robotic grasper was significantly improved over the standard handheld Doppler probe. MDP allowed for full range of motion of the robotic arms allowing the surgeon to easily scan vessels from a wide range of angles. No complications occurred.

#### Robotic assisted microscopic vasectomy reversal

A number of groups have developed robotic assisted techniques to perform Robotic assisted microscopic vasectomy reversal (RAVV) in animal and ex-vivo human models.[21–25] Some studies suggest that robotic assisted reversal may have advantages over microsurgical reversal in terms of ease of performing the procedure and improved patency rates.[23,24] A few groups have actually performed human robotic assisted vasovasostomies using the initial DaVinci robotic system[26] (Intuitive Surgical, Sunnyvale, CA).

These efforts have been recently confirmed in human RAVV cases performed using the new DaVinci S system.[27] We performed a prospective control study comparing initial results for RAVV in 20 human cases compared to seven standard microscopic vasovasostomies (MVV) cases by a single fellowship trained microsurgeon from July 2007 to June 2009.[28] A three-layer 10-0 and 9-0 suture anastomosis was performed with up to 22 months follow-up (mean three months). Mean operative duration for the RAVV cases was 109 min and 128 min for MVV (*P* 0.09). At two months post-op, all patients were patent (defined as greater than 2 million motile sperm in ejaculate). Mean sperm count was 54 million in RAVV and 11 million in MVV (*P* 0.04). The use of robotic assistance in microsurgical vasovasostomy may have benefit over MVV in decreasing operative duration and significantly improving early semen analysis measures. Further evaluation and longer follow-up is needed to assess its clinical potential and the cost-benefit ratio.

#### Robotic assisted microscopic varicocelectomy

Although reports of robotic assisted laparoscopic intra-abdominal varicocelectomy have been published,[29] there are a number of publications that suggest that microscopic subinguinal varicocelectomy (MVx) may provide superior outcomes compared to intra-abdominal varicocelectomy.[30–33] Shu, Wang *et al*., were the first to publish on robotic assisted microsurgical subinguinal varicocelectomy (RAVx).[34] They compared standard microsurgical to robotic assisted varicocelectomy and found that the robotic approach provided advantages in terms of slightly decreasing operative duration and complete elimination of surgeon tremor.

A review of our initial 46 human cases from June 08-September 2009 is as follows. Mean operative duration per side was 38 min (25-80). Indications for the procedures were: four for azoospermia, 25 for oligospermia and 17 for testicular pain (had failed all other conservative treatment options). Three-month follow-up is available for 23 patients: 76% (13 patients) with oligospermia had a significant improvement in sperm counts (three achieved pregnancy), and three with azoospermia remain unchanged. For testicular pain patients: 88% (15/17 patients) had complete resolution of pain. One recurrence or persistence of a varicocele occurred (by physical and ultrasound exam) and one patient developed a small postoperative hydrocele. The fourth robotic arm allowed the surgeon to control one additional instrument during the cases decreasing reliance on the microsurgical assistant. Robotic assisted microsurgical subinguinal varicocelectomy appears to be feasible. There are potential advantages in decreasing operative duration and improving surgeon efficiency. Further prospective randomized control trials are needed to define the benefit of robotic assistance in this procedure over standard microsurgery.

#### Robotic assisted microscopic denervation of the spermatic cord

Recent studies by Levine *et al*.,[35] and Oliveira *et al*.,[36] have shown that microscopic denervation of the spermatic cord is an effective treatment option for men with chronic testicular pain. Our group has been developing a robotic assisted microsurgical approach for the denervation of the spermatic cord Robotic assisted microscopic denervation of the spermatic cord (RMDSC) to assess if there may be any potential benefit over the standard microscopic technique.

A review of our initial 62 RMDSC cases from October 2008-November 2009 was performed (mean follow-up three months). Selection criteria for patients were as such: chronic testicular pain (>three months), failed standard pain management treatments and negative neurologic and urologic workup. A robotic assisted subinguinal or inguinal approach was utilized based on the location of pain. Pain was assessed utilizing a standardized validated tool (PIQ-6). The mean operative duration was 37 min (5-95); 87% (54/62) patients had a decrease in their pain (no change in eight patients). In the patients that had a response, by one month post-op, 69% had a PIQ-6 score of ≤50 (pain had no impact on the patient’s wellbeing and ability to function in everyday life). The effect was durable up to 6-12 months in the few patients that had follow-up to that point. The fourth robotic arm allowed the surgeon to control one additional instrument leading to less reliance on the microsurgical assistant.

#### Robotic assisted microscopic testicular artery re-anastomosis

During a robotic assisted denervation of the spermatic cord in a patient with chronic testicular pain, an inadvertent injury to one of the three testicular arteries occurred. Real-time intra-operative Doppler mapping of the testicular arteries had been performed during this case and helped to identify the injury immediately. The two ends of the artery were tied and the denervation procedure was completed. Microvascular atraumatic clamps were placed on both ends of the artery, the injured segment of artery was excised and a robotic assisted microsurgical vascular anastomosis was performed with eight interrupted 8-0 prolene sutures. The clamps were removed and arterial flow was confirmed distally with Doppler evaluation. The operative duration (skin to skin) for the entire procedure – denervation and artery repair – was 95 min. The patient was admitted overnight for observation and then discharged the next morning without any further complications. At six months follow-up, there does not appear to be any testicular atrophy or testicular pain. We will be following this patient to assess long-term outcomes. This case illustrates the capability of the robotic microsurgical platform to not only perform the desired procedure, but also as an additional tool in the microsurgeon’s arsenal in repairing injuries that occur during procedures.

## DISCUSSION

Let us consider the impact of robotic assistance during laparoscopic radical prostatectomy in urology.[37] Pure laparoscopic prostatectomy was a very technically demanding procedure and was only being performed in a few select high-volume centers. With the introduction of the da Vinci Robotic system (Intuitive Surgical, Sunnyvale, CA), robotic assisted laparoscopic prostatectomy is now available at several smaller volume centers.[37] Whether the quality of the procedure offered at smaller centers differs from the high-volume center is beyond the scope of this manuscript. What we would like to emphasize is that access to this procedure has been tremendously improved.[37]

Based on our institutional experience, the primary fellowship-trained microsurgeon (SP) in the above mentioned robotic assisted microsurgical studies was able to increase surgical throughput with the use of robotic assistance (able to perform six microsurgical cases a day, up from two or three microsurgical cases a day). The time for robotic setup and room turnover was similar to that for microscopic setup. The disposable costs per case for robotic versus microscopic were similar, but what was significantly different was the initial capital investment in the robot ($2,000,000) versus the microscope ($250,000). As we approach a more cost-conscious medical climate, this may be a limiting factor in the adoption of robotic assisted microsurgery unless pricing of the robotic system would decrease and/or savings in increased operative efficiency can compensate for this added cost.

The cost-benefit ratio is likely to be a major driving force in the development of further robotic instrumentation and applications. But if history is a reliable predictor of future growth, the pace of development is likely to continue. As the technology is based upon intelligent electronics, perhaps Moore’s Law might apply and future growth will increase exponentially.[3]

## CONCLUSIONS

Robotic instrumentation is likely to continue to improve and expand the range of applications in various areas within urology. Robotic applications in a range of areas within urology including microsurgery have the potential to grow. To understand how we will deal with the coming maelstrom of technologic wonders, we could turn to history. Winston Churchill once advised, “The further backwards you look, the further forward you see.” The relevance of looking at the foundation of modern robotics is to begin to understand technology and the changes that will affect all aspects of our civilization and not just the way we practice urology. The history of robotics is almost as intriguing as the robots themselves, almost.
